# Effect of Chitosan Coating on the Postharvest Quality and Antioxidant Enzyme System Response of Strawberry Fruit during Cold Storage

**DOI:** 10.3390/foods4040501

**Published:** 2015-09-29

**Authors:** Milena Petriccione, Francesco Mastrobuoni, Maria Silvia Pasquariello, Luigi Zampella, Elvira Nobis, Giuseppe Capriolo, Marco Scortichini

**Affiliations:** Consiglio per la ricerca in agricoltura e l’analisi dell’economia agraria (CREA)-Unità di ricerca per la Frutticoltura, Via Torrino, 3-81100 Caserta, Italy; E-Mails: f.mastrobuoni@entecra.it (F.M.); silviapasquariello@libero.it (M.S.P.); luigizampella@live.it (L.Z.); elviranobis@hotmail.it (E.N.); giuseppe.capriolo@entecra.it (G.C.); marco.scortichini@entecra.it (M.S.)

**Keywords:** strawberry, chitosan, cold storage, bioactive compounds, antioxidant enzyme, principal component analysis

## Abstract

The effectiveness of chitosan fruit coating to delay the qualitative and nutraceutical traits of three strawberry cultivars, namely “Candonga”, “Jonica” and “Sabrina”, as well as the effects of chitosan on antioxidant enzymes were evaluated. The fruits were coated with 1% and 2% chitosan solution and stored at 2 °C for nine days. Samples were taken every three days. Physico-chemical (weight loss, soluble solid content and titratable acidity) and nutraceutical (total polyphenol, anthocyanin, flavonoid, ascorbic acid content and antioxidant capacity) properties along with the enzymatic activity (catalase (CAT), ascorbate peroxidase (APX), polyphenol oxidase (PPO), guaiacol peroxidase (GPX) and lipoxygenase (LOX)) were evaluated. Chitosan treatment significantly reduced water loss and delayed the qualitative changes in color, titratable acidity and ascorbic acid content in dose- and cultivar-dependent manners. Additionally, changes in the total polyphenol, anthocyanin and flavonoid contents and the antioxidant capacity of chitosan-coated strawberry fruits were delayed. Chitosan coating enhanced the activity of some antioxidant enzymes, preventing flesh browning and reducing membrane damage. A global view of the responses of the three strawberry cultivars to chitosan coating and storage temperature was obtained using principal component analysis. Chitosan-coated fruit exhibited a slower rate of deterioration, compared to uncoated fruit in all tested cultivars.

## 1. Introduction

Strawberry (*Fragaria* × *ananassa* Duch.) is a “false fruit”, highly appreciated worldwide not only for its unique taste and distinct flavor, but also for its health benefits. Strawberries contain usual nutrients, such as minerals and vitamins, and a diverse range of anthocyanins, flavonoids and phenolic acids with biological properties, such as antioxidant, anticancer, anti-neurodegenerative and anti-inflammatory activities [1]. The phytochemical composition is influenced through genotype, environmental conditions and pre- and post-harvest factors [2,3].

However, strawberries have a short postharvest life with rapid spoilage, reflecting high susceptibility to mechanical injury, excessive texture softening, physiological disorders and infection through several pathogens during transport, storage and processing [4].

Several studies have demonstrated that the postharvest life of strawberries can be extended by different preservation techniques, such as refrigeration, synthetic chemical fungicides, modified atmosphere packaging, osmotic treatments, hypobaric treatments, heat treatments and edible coating [5,6,7,8,9,10]. In the last several years, edible coatings have been widely studied for the preservation of fruits and vegetables. Edible coating with semipermeable films might extend the postharvest life of strawberry through a reduction of moisture, gas exchange, respiration and oxidative reaction rates [11,12].

Chitosan, a deacetylated derivate of chitin, is a high molecular weight cationic linear polysaccharide composed of d-glucosamine and, to a lesser extent, *N*-acetyl-d-glucosamine with a β-1,4-linkage [13]. Chitosan is typically extracted from an abundant source of shellfish exoskeletons or the cell wall of some microorganisms and fungi [14]. Chitosan-based coatings are considered the best edible and biologically safe preservative coatings for different types of fruits, with functional advantages, such as slower respiration rates, extended storage periods, firmness retention and controlled microbial growth [4,15,16].

In strawberries, the effectiveness of some edible coatings based on chitosan [10,17,18], chitosan combined with essential oils [4], chitosan-beeswax [11], chitooligosaccharide [17], carboxymethyl cellulose and hydroxypropyl methylcellulose [19] has been tested.

To enlarge the knowledge of chitosan as a strawberry-coating compound, we evaluated the influence of two chitosan coating postharvest treatments on three strawberry cultivars based on changes in the total phenol, anthocyanin, flavonoid and ascorbic acid contents and the antioxidant capacity during cold storage. In addition, the effectiveness of the chitosan coating treatments on postharvest oxidative stress in strawberry fruit was also investigated.

## 2. Experimental Section

### 2.1. Fruit Samples

Three strawberry cultivars were selected: “Candonga”, “Jonica” and “Sabrina”. The fruits were randomly harvested at the commercial ripening stage and screened for uniformity and the absence of physical defects or decay. Subsequently, the strawberry fruits were randomly distributed into three groups prior to treatment. Chitosan (Iko Hydro, Rutigliano, Italy) with 90% deacetylation and a molecular weight of 360 kDa was prepared at two different concentrations, 1% and 2% (*w*/*v*) in an aqueous solution of acetic acid (0.5% *v*/*v*). The solution was warmed to 45 °C and stirred on a magnetic stirrer for complete dissolution of chitosan, adjusting its pH to 5.2 with NaOH. After cooling at 20 °C, the strawberry fruits were dipped in the chitosan solution for 60 s to allow the chitosan to adhere to the whole fruit surface to create a uniform film [20]. The fruits were dipped into the chitosan solution for 60 s to facilitate the chitosan adherence to the entire fruit surface to generate a uniform film. The samples were dried at room temperature, and 12 lots (100 g each) were prepared in small PET boxes per cultivar per treatment. The same number of lots was prepared with control fruits dipped in distilled water. The fruits were stored in a controlled chamber at 2 °C and 95% relative humidity and subsequently removed after 3, 6 and 9 days of cold storage. For each sampling date, three biological replicates per treatment per cultivar were prepared. All analyses were performed in triplicate.

### 2.2. Weight Loss, Firmness and Surface Color

Weight loss was calculated according to the weight of each sample before and after storage and expressed as the percentage weight loss compared to the initial weight.

Firmness was measured on two opposite sides of 15 fruits per cultivar per treatment using a digital penetrometer with a 5 mm-diameter probe, and the average value was expressed in Newton (N). The test conditions used for the measurement were 5 mm/s for pre- and post-test and 1 mm/s for the test speed with a penetrating distance of 5 mm into the fruit.

Skin color was assessed using a Minolta colorimeter (CR5, Minolta Camera Co., Japan) to determine the chromaticity values (L* (lightness), a (green to red) and b (blue to yellow)) on the opposite sides of 15 fruits per treatment. The hue angle (H*) was calculated using the chromaticity values a and b according to McGuire [21].

### 2.3. Total Soluble Solids Content and Titratable Acidity Content

The total soluble solids content (TSS, Brix) was determined for the flesh juice using a digital refractometer (Sinergica Soluzioni, DBR35, Pescara, Italy). The total acid content (TA) was determined after titrating 10 mL of flesh juice with 0.1 M NaOH, and the results were expressed as g citric acid/L.

### 2.4. Extraction and Measurements of Phenolic Compounds, Anthocyanins, Flavonoids and Total Antioxidant Capacity Assay

The methanol extracts for the different bioactive compound assays were obtained according to the method of Tomás-Barberán and Espín [22] with some modifications. Samples of 5 g of fresh weight each cut from 10 fruits were extracted with 25 mL of methanol (50% *v*/*v*). The homogenized samples from the methanol supernatants were subsequently centrifuged at 12,000× *g* for 15 min at 4 °C. The pellet was re-extracted using 25 mL of acetone (70% *v*/*v*) and centrifuged again at 12,000× *g* for 15 min at 4 °C. The resulting supernatants were mixed, filtered and subsequently used for the following assays.

The total phenol content in the strawberry fruits was determined using the Folin-Ciocalteu method [23], and the results are expressed as milligrams of gallic acid equivalents (GAE) per 100 grams fresh weight (FW) using gallic acid as a standard.

The total monomeric anthocyanins were estimated using the pH-differential method [24], and the results are expressed as cyanidin-3-glucoside equivalent (CGE) per 100 grams fresh weight. The absorbance was measured at 520 and 700 nm.

The total flavonoid content was determined using the aluminum chloride colorimetric method [25] with catechin as a standard. The total flavonoid content was expressed as milligrams of catechin equivalent (CE) per 100 grams fresh weight (FW).

The total antioxidant activity of strawberry fruit extracts was measured using 1,1-diphenyl-2-picryl-hydrazil (DPPH) according to the method of Brand-Williams, *et al.* [26] with some modifications. The assay was performed in a final volume of 1.5 mL in triplicate per sample. The percentage decrease in the DPPH concentration was calculated from the initial value after incubation for 15 min. A dose-response curve was generated, using Trolox as a standard, and the antioxidant activity was expressed as µmol Trolox equivalents (TE) per gram fresh weight (FW).

### 2.5. Ascorbic Acid Content

The ascorbic acid content was determined according the method of Malik and Singh [27], with some modifications. The strawberry fruits (2.5 g) were homogenized using 10 mL of 16% (*v*/*v*) metaphosphoric acid solution containing 0.18% (*w*/*v*) disodium ethylene diamine tetraacetic acid. The homogenate was centrifuged at 5000× *g* for 10 min, filtered and collected. The assay mixture contained 400 μL of extract, 200 μL of 3% metaphosphoric acid and 200 μL of diluted Folin’s reagent (1:5, *v*/*v*) in a final volume of 2 mL. After incubation for 10 min, the absorbance was measured at 760 nm using a UV-VIS spectrophotometer (Model V-630, JASCO, Japan). The ascorbic acid content was expressed as milligrams of ascorbic acid (AA) per 100 grams fresh weight (FW).

### 2.6. Enzyme Extraction and Activity Assays

#### 2.6.1. Catalase, Ascorbate Peroxidase and Guaiacol Peroxidase Activity

Total soluble proteins were extracted after re-suspending 1 g of frozen fruit tissue powder in 5 mL of extraction buffer containing 1 mL of 500 mM potassium phosphate buffer (pH 7.8), 500 μL of 10 mM sodium EDTA (pH 7), 5% (*w*/*w*) polyvinylpolypyrrolidone (PVPP) and 50 μL of 500 mM ascorbic acid (the ascorbic acid was only for APX enzyme extraction). The homogenate was centrifuged at 18,000× *g* for 10 min at 4 °C. The resulting supernatant was used to determine catalase, ascorbate peroxidase and guaiacol peroxidase activities. The protein content in all crude enzyme extracts examined was estimated using the Bradford assay [28], with bovine serum albumin as a standard.

Catalase (EC 1.11.1.6) (CAT) activity was assayed according to the method of Garcìa-Limones, *et al.* [29], with minor modifications. The reaction medium contained 150 μL of 500 mM potassium phosphate buffer (pH 7), 340 μL of 88 mM H_2_O_2_ and 200 µL of crude enzyme extract in a final volume of 1.5 mL. The reaction was initiated after the addition of H_2_O_2_, and the decrease in absorbance of peroxide was measured at 240 nm. The specific activity was expressed as µmol H_2_O_2_/min/mg protein.

Ascorbate peroxidase (EC 1.11.1.11) (APX) activity was determined according to Garcìa-Limones, *et al.* [29], with some modifications. The reaction mixture consisted of 300 μL of 500 mM potassium phosphate buffer (pH 7), 100 μL of 5 mM ascorbic acid, 6 μL of 88 mM H_2_O_2_, 100 μL of 10 mM sodium EDTA (pH 7) and 100 µL of crude enzyme extract in a final volume of 1.5 mL. The reaction was initiated after the addition of H_2_O_2_, and the oxidation of ascorbic acid was monitored at 290 nm. The specific activity was expressed as µmol ascorbate/min/mg protein.

Guaiacol peroxidase (EC 1.11.1.7) (GPX) activity was assayed according to Rao, *et al.* [30], with some modifications. The reaction mixture contained 200 μL of 500 mM potassium phosphate buffer pH 7, 15 μL of 10 mM sodium-EDTA (pH 7.0), 54 μL of 88 mM H_2_O_2_, 200 μL of 32 mM guaiacol and 350 µL of crude enzyme extract in a final volume of 1 mL. Guaiacol peroxidase activity was detected spectrophotometrically based on the formation of tetraguaiacol and the consequent increase in absorbance of this compound at 470 nm. One unit of enzyme activity was defined as the amount of enzyme that caused an increase in absorbance of 0.01 min. The specific enzyme activity was expressed as µmol tetraguaiacol/min/mg protein.

#### 2.6.2. Polyphenol Oxidase Activity

Polyphenol oxidase (PPO) activity was determined according to the method of Cao, *et al.* [31], with some modifications. A total of 2.5 g of fruit was homogenized in 5 mL of 200 mM sodium phosphate buffer (pH 6.5) containing 5% (*w*/*w*) PVPP. After incubation on ice for 1 h, the homogenate was centrifuged at 12,500× *g* for 10 min at 4 °C. The crude enzyme extract (100 µL) was subsequently incubated with 1.4 mL of 500 mM catechol in a final volume of 1.5 mL, and the increase in absorbance at 398 nm was monitored. The specific activity was expressed as µmol catechol/min/mg protein.

#### 2.6.3. Lipoxygenase Activity

Lipoxygenase (LOX) activity was quantified according to the method of Pérez, *et al.* [32], with slight modifications. The enzyme was extracted after re-suspending 1 g of frozen fruit tissue powder with 3 mL of extraction buffer (300 μL of 500 mM potassium phosphate buffer (pH 7.8), 300 μL of 10 mM sodium-EDTA (pH 7)) and 2% (*w*/*w*) PVPP. The reaction mixture consisted of 1.4 mL of 100 mM sodium phosphate buffer (pH 6), 50 μL of 5 mM linoleic acid sodium salt and 50 µL of crude enzyme extract in a final volume of 1.5 mL. The lipoxygenase activity was detected spectrophotometrically based on the formation of hydroperoxides and the resulting increase in absorbance at 234 nm. LOX activity was expressed as nmol hydroperoxides/min/g FW.

### 2.7. Malondialdehyde Content Determination

The malondialdehyde (MDA) content was evaluated according to the method of Peever and Higgins [33] with modifications. A total of 1 g of frozen fruit tissue was homogenized with 10 mL of extraction buffer (10% trichloroacetic acid, 0.25% thiobarbituric acid in 0.25 N HCl).

The samples were heated at 95 °C for 30 min and subsequently cooled on ice, except the blank, which was prepared using the same method without heating. Subsequently, all samples were centrifuged at 10,000× *g* for 10 min at 4 °C. Aliquots (1 mL) of the supernatant were used for spectrophotometric determination at 432, 532 and 600 nm. The MDA content was calculated according to Bao, *et al.* [34] and expressed as µmol/100g FW.

### 2.8. Statistical Analysis

The data are expressed as the mean ± standard deviation. To determine differences between uncoated 1% and 2% chitosan-coated fruit in each strawberry cultivar, one-way ANOVA and the least significant difference (LSD) test for mean comparisons were used. Differences at *p* < 0.05 were considered significant and are indicated with different letters. Correlations among the evaluated parameters were analyzed using Pearson’s correlations (*p* < 0.05 and *p* < 0.01). Principal component analysis (PCA) was applied to describe the relationship between the physical-chemical and nutraceutical traits and the enzymatic activities in order to identify the principal components contributing to the majority of the variation within the dataset. All analyses were performed using the SPSS software package, Version 20.0 (SPSS Inc., Chicago, IL, USA).

## 3. Results and Discussion

### 3.1. Effect of Chitosan Treatment on Weight Loss and Firmness

**Figure 1:**
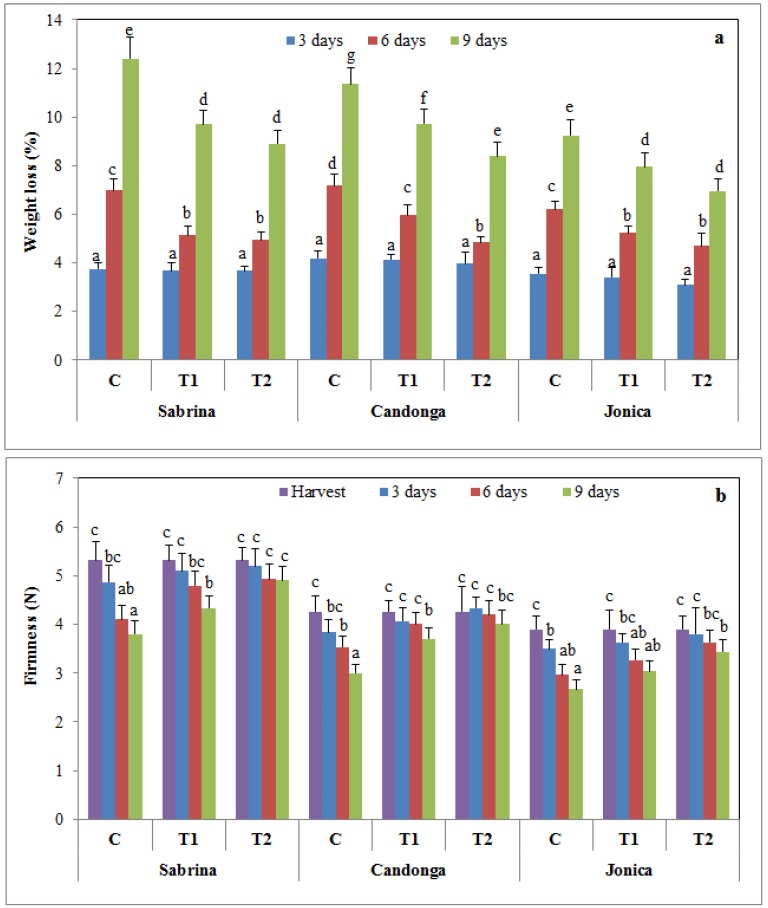
Changes in weight loss (**a**) and firmness (**b**) of strawberry cultivars during cold storage at 2 °C for nine days (C, control; T1, 1% chitosan coating; T2, 2% chitosan coating; error bars indicate the standard deviation).

The weight loss increased throughout the cold storage period in chitosan-coated and uncoated strawberry fruit with significant (*p* < 0.05) differences among and within the strawberry cultivars (Figure 1a). Chitosan coating limited fruit weight loss compared to uncoated fruit, and a better effect on delaying the weight loss of strawberries during the nine days of storage was observed for the 2% chitosan coating. At the end of the storage period (ninth day), the weight loss in strawberry coated with 2% chitosan was (6.97% ± 0.49%) in “Jonica”, followed by “Candonga” (8.40% ± 0.59%) and “Sabrina” (8.98 ± 0.53).

The loss of weight in fresh fruit primarily reflects the respiration rate and moisture evaporation between the fruit tissue and surrounding air, which are influenced by postharvest treatment and storage temperature [18]. Strawberry fruits are highly susceptible to a rapid loss of water due to the extremely thin skins of these fruits. These results are consistent with those of previous studies demonstrating that chitosan coating acts as a semipermeable barrier against oxygen, carbon dioxide and moisture, thereby reducing respiration and water loss and counteracting the dehydration and shrinkage of the fruit [11,20,35].

Firmness is an important physical parameter used to assess the quality of fruits during ripeness, storage and distribution [36]. At harvest, strawberry cultivars showed different firmness values that could be due to different lignin content. The firmness decreased in uncoated and chitosan-coated fruit during the storage period in a cultivar-dependent manner (Figure 1b). Treatment with 2% chitosan coating was more effective in maintaining the firmness of the strawberries compared to 1% chitosan coating, although the difference between these two treatments was not significant (*p* < 0.05) until Day 6 in all strawberry cultivars. After nine days of storage, the loss of firmness in uncoated fruit was 31.62% (“Jonica”), 29.69% (“Candonga”) and 28.52% (“Sabrina”), while strawberry cultivars coated with 2% chitosan showed reduction of firmness values of 11.96%, 6.25% and 7.84%, respectively (Figure 1b), indicating that chitosan postharvest treatment significantly inhibited the softening of strawberry fruit resulting from the degradation of the middle lamella of the cell wall of cortical parenchyma cells [37]. Previous studies have reported the beneficial effects of several coating applications, such as cactus mucilage [38], chitosan-oleic acid [39], chitosan in combination with calcium dips [18] and chitosan-beeswax [11], on the strawberry texture.

### 3.2. Effect of Chitosan Treatment on TSS, TA and Surface Color of Strawberry Fruit

The TSS values of chitosan-coated (1% and 2%) and uncoated fruits gradually increased during the nine days of cold storage in all strawberry cultivars (Table 1). At the end of cold storage, uncoated “Jonica” and “Sabrina” fruits exhibited significantly (*p* < 0.05) higher TSS compared to all other chitosan-coated fruits. These results are consistent with those of other studies concerning the effects of chitosan-coated treatment on different commodities, such as mango, guava, banana, papaya, guava and sweet cherry [20,40,41,42].

This increase could reflect the cell wall disassembly [43], the decrease in respiration rate and the increase in dry matter due to water loss [44]. The low increase in TSS values for the chitosan-coated fruits, from the three strawberry cultivars, might reflect changes in the internal atmosphere of the fruit, with a reduction in the O_2_ level and/or an increase in the CO_2_ level, which reduce the respiration rate and metabolic activity, such as the conversion of sugars into CO_2_ and H_2_O [45].

The TA estimates the organic acid contents of fleshy fruits, and in strawberry fruit, the main organic acids are citric and malic acid [46]. This trait is an important component of fruit organoleptic quality and is different in each cultivar. TA showed a significant decrease throughout cold storage in strawberry fruits, with lower values in uncoated fruit compared to chitosan-coated fruit (Table 1).

Previous studies have suggested that the higher acidity loss in uncoated fruits might reflect the use of organic acids as substrates for respiratory metabolism during storage [47,48]. Chitosan treatment plays an important role in delaying fruit ripening during cold storage, and chitosan-coated fruits showed a lower acidity loss, consistent with other studies on strawberry, peach, guava and litchi [49,50,51].

The TSS/TA ratio, the most important parameter in evaluating strawberry quality, determines fruit flavor harmony and consumer acceptability [52]. Chitosan-coated (1% and 2%) fruits exhibited a smaller increase in the TSS/TA ratio than uncoated fruits during cold storage, reflecting reduced ripening compared to uncoated strawberries (Table 1).

Surface color is an important index to evaluate the quality and ripening process in strawberry fruit; at harvest, three strawberry cultivars showed different color parameters (L* and H angle). These values decreased during cold storage, and the strawberry color darkened with a reduction in hue angle values. At nine days of cold storage, H values in uncoated fruit were 23.03 ± 0.41, 24.65 ± 0.17 and 25.92 ± 0.47 in “Sabrina”, “Candonga” and “Jonica”, respectively. Color changes, in H values, were less noticeable in chitosan-coated (1% and 2%) fruits. The L* value decreased with lower values in uncoated fruit than in chitosan-coated (1% and 2%) fruits at the end of the cold storage period (Table 1).

**Table 1:** Effect of chitosan treatments on lightness (L*), hue angle (H), soluble solid content (TSS), titratable acidity (TA) and the TSS/TA ratio of three strawberry cultivars tested as a function of cold storage (CS) period.

Cultivar		Days of CS	L*	H	TSS (°Brix)	TA (mg Citric Acid/L Juice)	TSS/TA
**Sabrina**	C	0	28.53 ± 1.08 ^e^	30.8 ± 1.12 ^f^	8.37 ± 0.32 ^a^	7.93 ± 0.20 ^f^	1.05 ± 0.06 ^a^
		3	27.25 ± 0.61 ^d,e^	27.71 ± 1.46 ^d,e^	9.93 ± 0.06 ^b^	6.60 ± 0.27 ^e^	1.50 ± 0.05 ^b^
		6	24.29 ± 0.79 ^a^	26.7 ± 1.32 ^c,d^	11.93 ± 0.11 ^d,e^	5.10 ± 0.10 ^b^	2.34 ± 0.05 ^e^
		9	24.50 ± 0.99 ^a^	23.03 ± 0.41 ^a^	13.07 ± 0.81 ^g^	4.17 ± 0.15 ^a^	3.14 ± 0.14 ^g^
	T1	0	28.53 ± 1.08 ^e^	30.08 ± 1.13 ^f^	8.37 ± 0.32 ^a^	7.93 ± 0.20 ^f^	1.05 ± 0.06 ^a^
		3	27.49 ± 1.01 ^e^	28.02 ± 0.05 ^d,e^	9.67 ± 0.25 ^b^	6.67 ± 0.25 ^e^	1.45 ± 0.09 ^b^
		6	24.70 ± 0.79 ^a,b,c^	27.11 ± 0.56 ^c,d^	11.33 ± 0.15 ^d^	5.57 ± 0.15 ^c^	2.03 ± 0.09 ^d^
		9	24.60 ± 0.82 ^a,b^	24.53 ± 0.56 ^b^	12.57 ± 0.59 ^f,g^	4.97 ± 0.15 ^b^	2.53 ± 0.04 ^f^
	T2	0	28.53 ± 1.08 ^e^	30.08 ± 1.13 ^f^	8.37 ± 0.32 ^a^	7.93 ± 0.20 ^f^	1.05 ± 0.06 ^a^
		3	27.68 ± 0.27 ^e^	29.08 ± 0.50 ^c,f^	9.33 ± 0.15 ^b^	6.77 ± 0.32 ^e^	1.38 ± 0.09 ^b^
		6	25.89 ± 0.20 ^b,c,d^	27.61 ± 0.21 ^d^	10.67 ± 0.25 ^c^	6.10 ± 0.10 ^d^	1.75 ± 0.06 ^c^
		9	26.04 ± 0.47 ^c,d^	25.97 ± 0.02 ^b,c^	12.07 ± 0.20 ^e,f^	5.27 ± 0.15 ^b,c^	1.80 ± 0.65 ^e^
**Candonga**	C	0	26.83 ± 0.68 ^e^	28.92 ± 0.12 ^f^	8.57 ± 0.15 ^a^	11.03 ± 0.20 ^g^	0.78 ± 0.01 ^a^
		3	24.59 ± 0.42 ^d^	28.29 ± 0.36 ^e^	9.63 ± 0.20 ^c,d^	10.17 ± 0.15 ^e^	0.94 ± 0.03 ^c^
		6	23.04 ± 0.09 ^c^	25.39 ± 0.35 ^b^	10.20 ± 0.20 ^e,f^	9.00 ± 0.20 ^c^	1.13 ± 0.04 ^e^
		9	21.06 ± 0.06 ^a^	24.65 ± 0.17 ^a^	10.90 ± 0.60 ^g^	8.00 ± 0.10 ^a^	1.37 ± 0.09 ^g^
	T1	0	26.83 ± 0.68 ^e^	28.92 ± 0.12 ^f^	8.57 ± 0.15 ^a^	11.03 ± 0.20 ^g^	0.78 ± 0.01 ^a^
		3	25.03 ± 0.05 ^d^	28.43 ± 0.27 ^e^	9.27 ± 0.11 ^b,c^	10.67 ± 0.15 ^f^	0.88 ± 0.01 ^b^
		6	23.13 ± 0.02 ^c^	25.94 ± 0.61 ^c^	9.90 ± 0.10 ^d,e^	9.77 ± 0.15 ^d^	1.03 ± 0.005 ^d^
		9	21.07 ± 0.25 ^b^	24.73 ± 0.26 ^a^	10.80 ± 0.45 ^g^	8.47 ± 0.45 ^b^	1.27 ± 0.01 ^f^
	T2	0	26.83 ± 0.68 ^e^	28.92 ± 0.13 ^f^	8.57 ± 0.15 ^a^	11.03 ± 0.20 ^g^	0.78 ± 0.01 ^a^
		3	25.15 ± 0.07 ^d^	28.54 ± 0.092 ^e,f^	9.10 ± 0.10 ^b^	10.87 ± 0.15 ^f,g^	0.83 ± 0.02 ^b^
		6	24.63 ± 0.18 ^d^	26.78 ± 0.21 ^d^	9.57 ± 0.15 ^c,d^	10.00 ± 0.20 ^d,e^	0.95 ± 0.04 ^c,d^
		9	21.85 ± 0.10 ^b^	25.53 ± 0.11 ^b,c^	10.57 ± 0.15 ^f,g^	9.17 ± 0.15 ^c^	1.15 ± 0.02 ^e^
**Jonica**	C	0	28.87 ± 0.32 ^e^	30.57 ± 0.15 ^e^	13.00 ± 0.10 ^a^	8.43 ± 0.15 ^f^	1.54 ± 0.04 ^a^
		3	27.17 ± 0.06 ^d^	30.18 ± 0.13 ^e^	14.10 ± 0.10 ^c,d^	7.60 ± 0.26 ^d^	1.85 ± 0.07 ^c^
		6	22.60 ± 0.10 ^b^	26.18 ± 0.29 ^a,b^	14.73 ± 0.11 ^e,f^	6.70 ± 0.26 ^b^	2.2 ± 0.07 ^e^
		9	22.01 ± 0.02 ^a^	25.92 ± 0.47 ^a^	16.17 ± 0.29 ^g^	6.10 ± 0.10 ^a^	2.65 ± 0.08 ^g^
	T1	0	28.87 ± 0.32 ^e^	30.57 ± 0.15 ^e^	13.00 ± 0.10 ^a^	8.43 ± 0.15 ^f^	1.54 ± 0.03 ^a^
		3	27.41 ± 0.14 ^d^	30.47 ± 0.20 ^e^	13.87 ± 0.15 ^b,c^	7.97 ± 0.15 ^e^	1.74 ± 0.04 ^b^
		6	22.93 ± 0.04 ^b^	27.63 ± 0.11 ^d^	14.43 ± 0.20 ^d,e^	7.10 ± 0.10 ^c^	2.03 ± 0.02 ^d^
		9	22.92 ± 0.03 ^b^	26.63 ± 0.08 ^b,c^	15.43 ± 0.60 ^g^	6.63 ± 0.11 ^b^	2.32 ± 0.12 ^f^
	T2	0	28.87 ± 0.32 ^e^	30.57 ± 0.15 ^e^	13.00 ± 0.10 ^a^	8.43 ± 0.15 ^f^	1.54 ± 0.03 ^a^
		3	28.55 ± 0.50 ^e^	30.53 ± 0.41 ^e^	13.50 ± 0.27 ^b^	8.07 ± 0.15 ^e^	1.67 ± 0.01 ^b^
		6	23.44 ± 0.16 ^c^	27.90 ± 0.56 ^d^	14.27 ± 0.16 ^c,d^	7.50 ± 0.10 ^d^	1.9 ± 0.04 ^c^
		9	22.99 ± 0.02 ^b^	26.74 ± 1.94 ^c^	15.10 ± 0.37 ^f,g^	7.20 ± 0.10 ^c^	2.09 ± 0.03 ^d^

Statistical comparisons were made within each cultivar. Means followed by the same letter do not differ significantly at *p* < 0.05 (LSD test). C, uncoated fruit; T1, chitosan-coated fruit (1%); T2, chitosan-coated fruit (2%).

### 3.3. Effect of Chitosan Treatment on Nutraceutical Compounds and Antioxidant Activity

**Figure 2:**
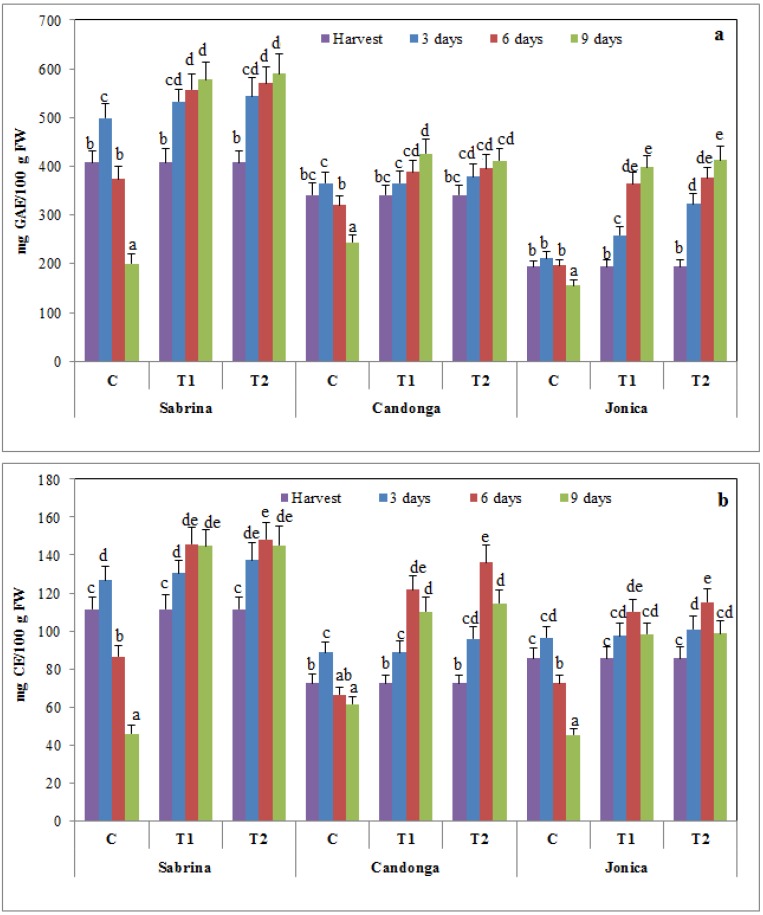
Changes in polyphenols (**a**) and flavonoids (**b**) content of strawberry cultivars during cold storage at 2 °C for nine days (error bars indicate standard deviation).

At harvest, the total phenolics content significantly differed (*p* ≤ 0.05) among the analyzed strawberry cultivars, with lower values in “Jonica” (194.7 ± 6.9 mg GAE/100 g FW) than in “Candonga” and “Sabrina” (341.6 ± 8.9 mg GAE/100 g FW and 407.3 ± 10.6 mg GAE/100 g FW, respectively) (Figure 2a).

**Figure 3:**
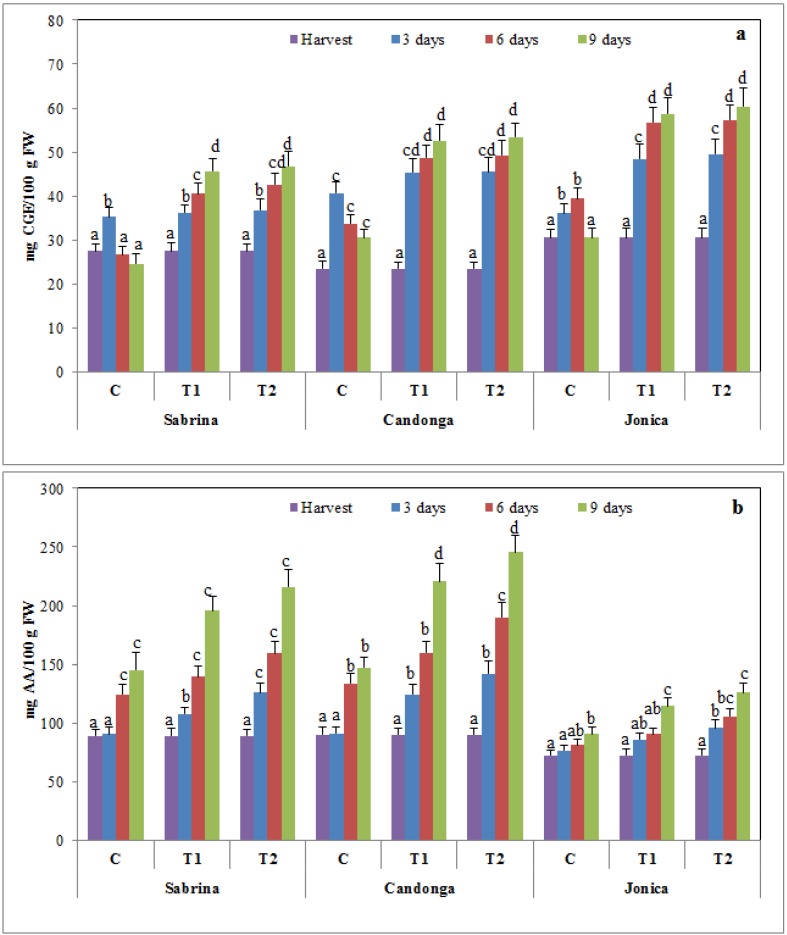
Changes of the total anthocyanins (**a**) and ascorbic acid (**b**) content of strawberry cultivars during cold storage at 2 °C for nine days (error bars indicate standard deviation).

The total phenolics content continuously increased in chitosan-coated fruit, and no effect of the chitosan concentration was observed. However, in uncoated fruit, a slight increase in the total phenolics content was observed until three days of cold storage, followed by a cultivar-dependent decrease (Figure 2a). Therefore, chitosan-coated fruit also maintained higher flavonoid and anthocyanin contents than uncoated fruit at the end of cold storage (Figure 2b and Figure 3a). These results are consistent with previous studies demonstrating that chitosan treatment improved the nutraceutical properties of strawberry fruits, maintaining high levels of phenols, anthocyanins and flavonoids during postharvest [10], further suggesting that chitosan treatment delays fruit senescence and enhances the phytochemical content during storage.

Several studies demonstrated that pre- and post-harvest application of external elicitors, such as chitosan, to vegetative tissue can trigger plant resistance [16]. Furthermore, chitosan activates the key enzyme in the phenol synthesis pathway, such as phenylalanine ammonia lyase (PAL), and increases total polyphenols in several fruits [16].

Ellagitannins, hydrolysable tannins present only in berries and nuts, are an important group of phenolic compounds in strawberry [53], and these fruits also contain high concentrations of condensed tannins, such as proanthocyanidin [54]. The phenolic compounds present in lower concentrations in strawberries include flavonols, *i.e.*, glycosides of quercetin and kaempferol, esters of hydroxycinnamic acids, particularly of *p*-coumaric acid, and ellagic acid and ellagic acid glycosides [54,55].

In strawberry fruits, the main anthocyanins include pelargonidin-3-glucoside, while pelargonidin-3-rutinoside and cyanidin-3-glucoside are present as minor components. The concentration and composition of these compounds, responsible for the bright red color of the berries, are important for the sensory quality and health benefits of the strawberry [56].

The ascorbic acid content increased in a cultivar-dependent manner throughout storage time in uncoated and coated fruit. “Candonga” showed the highest acid ascorbic content compared to “Sabrina” and “Jonica”. Chitosan treatment improved the increase during cold storage, with the highest values observed for the three strawberry cultivars coated with 2% chitosan coating (Figure 3b).Cordenunsi, *et al.* [43] demonstrated that ascorbic acid synthesis in strawberries occurs during the storage period, and temperature affects ascorbic acid synthesis. The results of the present study suggest that genotypes can also influence ascorbic acid synthesis during postharvest. Furthermore, the higher level of ascorbic acid in chitosan-coated fruit might reflect the low oxygen permeability, which reduced the activity of the enzymes involved in the oxidation of ascorbic acid [57].

**Figure 4:**
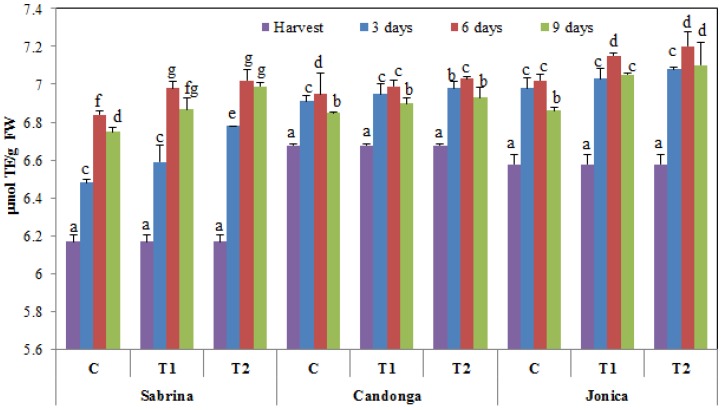
Changes in total antioxidant activity of strawberry cultivars during cold storage at 2 °C for nine days (error bars indicate standard deviation).

In the present study, the antioxidant capacity, measured using the DPPH assay, increased in strawberry fruit within the first six days and subsequently decreased; chitosan-coated fruit showed higher values compared to uncoated fruit (Figure 4).

Thus, chitosan coating, as an edible coating for strawberries, imparts beneficial effects in terms of the maintenance of nutraceutical compounds and antioxidant activity in a dose-dependent manner.

### 3.4. Effect of Chitosan Treatment on Antioxidant Enzymes

Postharvest oxidative stress occurs during fruit storage, causing an imbalance between the production and removal of reactive oxygen species (ROS), such as H_2_O_2_, O_2_^−^ and hydroxyl radicals, from the tissues. The protection of fruit cells from oxidative injury depends on the level of antioxidant enzymes, such as catalase (CAT), peroxidase (GPX) and superoxide dismutase (SOD), which scavenge ROS and prevent harmful effects [58,59].

**Figure 5:**
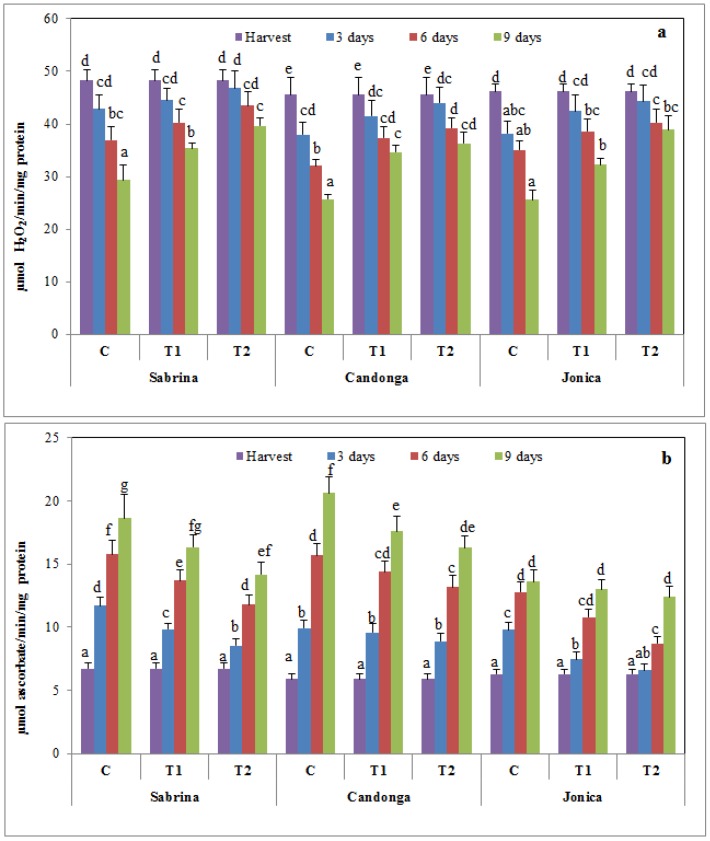
Changes in catalase (CAT) (**a**) and ascorbate peroxidase (APX) (**b**) activity of strawberry cultivars during cold storage at 2 °C for nine days (error bars indicate standard deviation).

CAT catalyzes the decomposition of hydrogen peroxide to water and oxygen, and this enzyme is commonly found in nearly all organisms exposed to oxygen [60]. CAT activity in strawberry fruits decreased during cold storage, but this decrease was higher in uncoated fruit than in coated fruits. Chitosan treatment suppressed this reduction in a dose-dependent manner (Figure 5a). These results are consistent with those of previous studies on guava, sweet cherry and strawberry fruits [10,42,57,61]. Thus, chitosan treatment plays an important role in oxidation resistance, improving CAT activity.

Ascorbate peroxidase (APX) catalyzes the reduction of hydrogen peroxide to water using ascorbate as an electron donor. Chitosan treatment reduced the increase in APX activity in a dose-dependent manner (Figure 5b).

This result suggested that chitosan treatment induced a different response on two H_2_O_2_ scavenging enzymes. As indicated by Davletova, *et al.* [62], CATs might be responsible for the removal of ROS during stress, and APX might be responsible for the fine-tuning of ROS during signaling.

### 3.5. Effect of Chitosan Treatment on Membrane Damage

Lipoxygenase (LOX) activity and malondialdehyde (MDA) content are used as measurements of the loss of membrane integrity in response to postharvest oxidative stress during storage [61].

**Figure 6:**
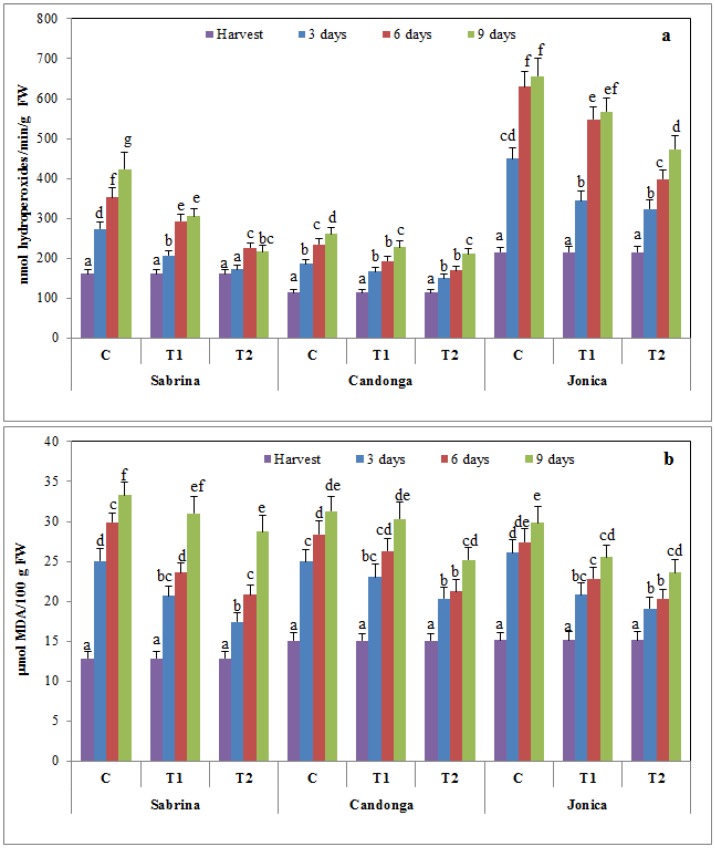
Changes in lipoxygenase activity (LOX) (**a**) and malondialdehyde content (MDA) (**b**) of strawberry cultivars during cold storage at 2 °C for nine days (error bars indicate standard deviation).

LOX activity increased during the 9 days of cold storage, suggesting that the dioxygenation of polyunsaturated fatty acids produces toxic hydroperoxy fatty acids and consequent membrane damage (Figure 6a). Chitosan-coated fruit showed significantly lower LOX activity compared to the control fruit in all of the analyzed cultivars, with differences between the two chitosan concentrations tested. In chitosan-coated fruit, “Jonica” showed values significantly (*p* < 0.05) higher than “Sabrina” and “Candonga”, implying greater preservation of membrane integrity.

The MDA content in uncoated fruit was higher than that in chitosan-treated fruit, which showed statistically lower values in a dose-dependent manner (Figure 6b).

These results suggest that chitosan is a promising tool for preventing postharvest oxidative damage during cold storage. Chitosan coating creates a barrier to the oxygen responsible for lipid peroxidation, thereby preserving the maintenance of membrane integrity [42,63].

### 3.6. Effect of Chitosan Treatment on Enzymatic Browning

**Figure 7:**
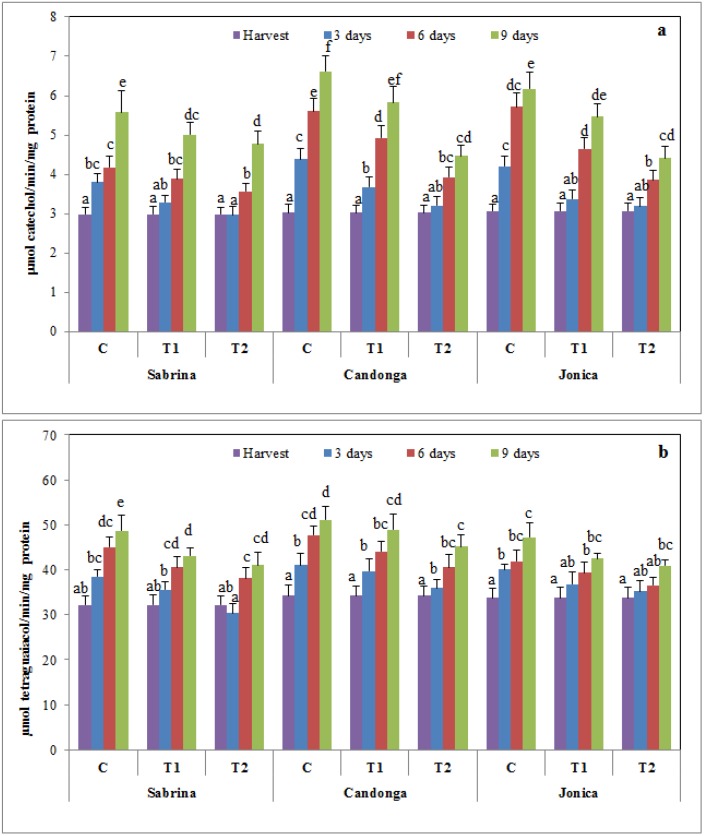
Changes in polyphenol oxidase (PPO) (**a**) and guaiacol peroxidase (GPX) (**b**) activity of strawberry cultivars during cold storage at 2 °C for nine days (error bars indicate standard deviation).

Polyphenol oxidases and peroxidases catalyze the reactions involved in enzymatic browning, the most important color reaction affecting fruits and vegetables [64]. Strawberry fruit showed an increase in PPO and GPX activity during the cold storage period, with higher values in uncoated fruit. Chitosan treatment induced a significant (*p* < 0.05) delay in PPO and GPX activity in a dose-dependent manner (Figure 7).

The inhibitory effect of chitosan treatment on PPO activity likely reflects low O_2_ availability, consistent with a previous study on sweet cherry [61]. Furthermore, the prolonged storage of chitosan-coated strawberry fruit increased the total phenolic content, likely reflecting lower PPO and GPX activity.

Similarly, inhibited GPX and PPO activities have been observed in response to alternative technologies used to improve the postharvest life of different fruit [65].

### 3.7. Principal Component Analysis

PCA is a valid tool to monitor the postharvest life of fruit, as previously demonstrated in other studies [36,66]. PCA was applied to evaluate the effectiveness of chitosan on the three strawberry cultivars during the cold storage period after analyzing the activity of qualitative traits and some enzymes correlated with oxidative stress, enzymatic browning and membrane damage. The cross-validation technique demonstrated that two principal components are necessary to explain the total variability of the traits analyzed. The eigenvalues of the covariance matrix showed that the set of the two principal components (PCs) accounted for 70.6% of the total variance in the dataset with respect to cold storage. PC1 explained 48.8% of the variance in the dataset, whereas PC2 explained an additional 21.8% of the variance. The weight loss, TSS/TA, anthocyanin and ascorbic acid contents, antioxidant activity, MDA content and APX, GPX and PPO activity were positively correlated with PC1, whereas the TA, L*, hue angle and CAT activity were negatively correlated. PC2 was only positively correlated with firmness and the polyphenol and flavonoid contents, while TSS and LOX were negatively correlated.

**Figure 8:**
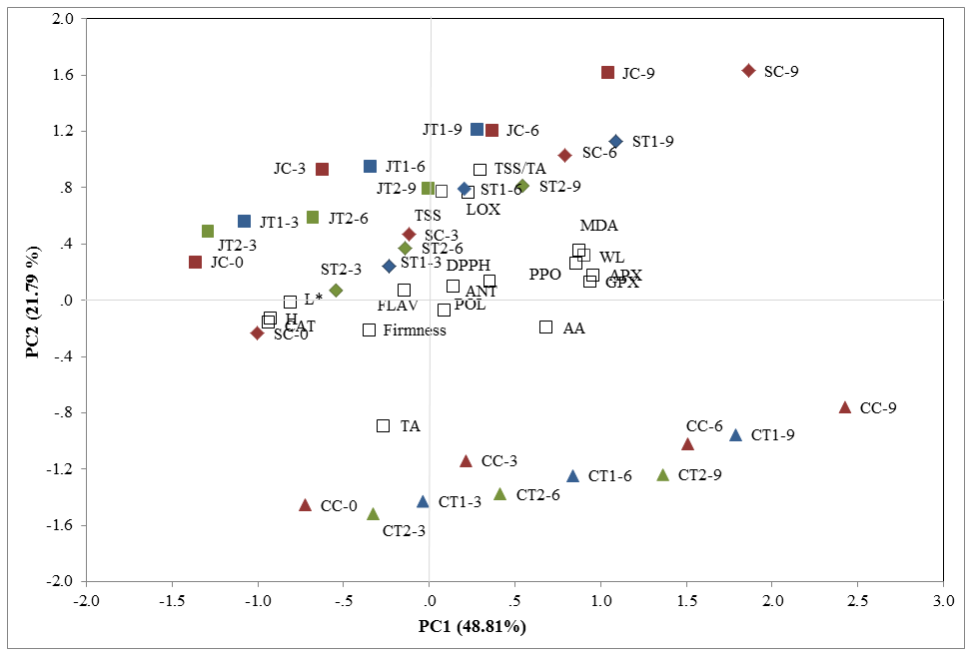
2D-principal component analysis plot of the pomological, nutraceutical and enzymatic attributes in the three strawberry cultivars “Sabrina” (S), “Candonga” (C) and “Jonica” (J) at harvest (C0), 3 (C-3: uncoated fruit; T1-3 chitosan-coated (1%) fruit; T2-3 chitosan-coated (2%) fruit), 6 (C-6: uncoated fruit; T1-6 chitosan-coated (1%) fruit; T2-6 chitosan-coated (2%) fruit) and 9 (C9: uncoated fruit; T1-9 chitosan-coated (1%) fruit; T2-9 chitosan-coated (2%) fruit) days of cold storage (WL: weight loss; TSS: total soluble solid content; TA: total titratable acidity; TSS/TA: TSS/TA ratio; L: L* value; H: hue angle; POL: polyphenol content; FLAV: flavonoid content; AA: acid ascorbic content; DPPH: radical scavenging activity; CAT: catalase; APX: ascorbate peroxidase; GPX: guaiacol peroxidase; LOX: lipoxygenase; PPO: polyphenol oxidase; MDA: malondialdehyde content).

Differences among the uncoated and chitosan-coated fruits with respect to the cold storage period and different strawberry cultivars are shown in a 2D-plot (Figure 8). Different behaviors in strawberry cultivars during cold storage were observed. The plots showed that more “Candonga” fruits were located in the lower axis, while “Sabrina” and “Jonica” were positioned in the upper axis. The uncoated fruit showed a higher shift in different PCs, suggesting wide changes in qualitative and enzymatic traits during storage in a cultivar-dependent manner. Chitosan-coated fruit exhibited a slower rate of deterioration, with a lower shift in different PCs in a dose-dependent manner. Notably, coated fruit (2%) at nine days of cold stage was comparable to uncoated fruit at six days of cold stage in all tested cultivars. Chitosan treatment preserved postharvest oxidative stress and qualitative changes, thereby prolonging cold storage for three more days.

## 4. Conclusions

Chitosan coating is a valid tool to improve the postharvest life of strawberry cultivars. Indeed, chitosan coatings exhibit film-forming properties on fruit surfaces and can be used as protective barriers to reduce respiration and transpiration rates and to suppress color changes, thereby improving fruit quality.

Chitosan coating positively influenced the physical-chemical and nutraceutical traits of the three strawberry cultivars analyzed, reduced postharvest oxidative stress through the enhancement of antioxidant enzyme activities that reduce oxidative damage via the regulation of ROS metabolism and delayed fruit browning through the inhibition of PPO and GPX activities. The improvement in storage and delays in senescence appearance were confirmed through a significant reduction in LOX activity and MDA content in the three strawberry cultivars. PCA showed the different behaviors of the three strawberry cultivars facilitating the monitoring of the qualitative and enzymatic decay rate in dose- and cultivar-dependent manners.
